# A Data-Driven Approach to Quantify and Measure Students’ Engagement in Synchronous Virtual Learning Environments

**DOI:** 10.3390/s22093294

**Published:** 2022-04-25

**Authors:** Xavier Solé-Beteta, Joan Navarro, Brigita Gajšek, Alessandro Guadagni, Agustín Zaballos

**Affiliations:** 1 Research Group in Internet Technologies & Storage, La Salle Campus Barcelona, Universitat Ramon Llull, Quatre Camins 30, 08022 Barcelona, Spain; xavier.sole@salle.url.edu (X.S.-B.); zaballos@salleurl.edu (A.Z.); 2 Fakulteta za Logistiko, Univerza v Mariboru, 3000 Celje, Slovenia; brigita.gajsek@um.si; 3 ValueDo srl, Via Filippo Corridoni 91, 50134 Florence, Italy; aleguadagni@gmail.com

**Keywords:** student engagement, virtual learning environments, digital interactions

## Abstract

In face-to-face learning environments, instructors (sub)consciously measure student engagement to obtain immediate feedback regarding the training they are leading. This constant monitoring process enables instructors to dynamically adapt the training activities according to the perceived student reactions, which aims to keep them engaged in the learning process. However, when shifting from face-to-face to synchronous virtual learning environments (VLEs), assessing to what extent students are engaged to the training process during the lecture has become a challenging and arduous task. Typical indicators such as students’ faces, gestural poses, or even hearing their voice can be easily masked by the intrinsic nature of the virtual domain (e.g., cameras and microphones can be turned off). The purpose of this paper is to propose a methodology and its associated model to measure student engagement in VLEs that can be obtained from the systematic analysis of more than 30 types of digital interactions and events during a synchronous lesson. To validate the feasibility of this approach, a software prototype has been implemented to measure student engagement in two different learning activities in a synchronous learning session: a masterclass and a hands-on session. The obtained results aim to help those instructors who feel that the connection with their students has weakened due to the virtuality of the learning environment.

## 1. Introduction

Influencing student engagement is an important concern for instructors, as it positively impacts the quality of education and learning [1]. In fact, engagement in education has shown to be a good indicator of the willingness of students to invest a considerable amount of mental effort and persistence to construct the necessary understanding of new concepts taught in learning environments [2,3,4]. To keep students fully engaged in the learning process, instructors influence, or even dictate, different learning activities to later perceive how they impact on the students, interpret what it is happening in the classroom according to their knowledge and training experience, and finally steer more activities in the desired direction [5]. In fact, this teaching experience plays a crucial role in enabling instructors to unconsciously, constantly, and automatically infer student engagement and dynamically adapt the training activities according to the perceived student reactions. Similarly, when the instructor does not follow student response (i.e., feedback), he/she gradually loses contact with the students. This leads to well-known situations (see Tinto’s Model of Student Departure [6]) where some students start skipping lectures, or become busy with other things during lectures, or do not follow the content presentation and, thus, can less and less actively follow further lectures. According to the literature, there is a consequent connection between student (loss of) engagement and their academic performance [7,8,9]. Therefore, one of the instructor’s most prominent goals when designing and conveying a training lesson is to keep students engaged to make sure that the delivered content is successfully assimilated. Hence, student engagement can be best seen as a Key Performance Indicator of the whole learning process [10].

So far, a large number of strategies to foster student engagement has been proposed, ranging from promoting task relevance and unveiling hidden value for student careers [4] to forcing students to explicitly interact with the learning environment [11], including supporting lessons with alternative activities and/or methods of instruction such as gamification (also referred to as Game-Based Learning) [9,12,13], peer instruction [14], or group activities [15]. These strategies have been elaborated and (are being) refined as a result of decades of teaching experience. Although it has been shown that these strategies contribute to an increase in student engagement, there is still a lot of research being conducted on how to reliably quantify and measure student engagement in an unbiased way. Indeed, the conception of student engagement has evolved over time according to the evolution of society and technology [16]. For instance, early definitions of student engagement in the 1980s limited the scope of engagement to student participation in school-offered activities [16]. More precisely, Astin [2] proposed to define student involvement (i.e., engagement) as the amount of physical and psychological energy that students devote to the academic experience. Presently, the most widely accepted definitions come from recent studies in North America that consider engagement as a metaconstruct that encompasses four components [16,17,18]:

- Academic: Associated with to which extent students are motivated to learn and do well in school. It can be measured with variables such as time on task, credits earned toward graduation, and homework completion.
- Behavioral: Associated with positive conduct, effort, participation in teaching and learning activities, and compliance with rules or norms. It can be measured with variables such as attendance, suspensions, voluntary classroom participation, and extracurricular participation.
- Emotional or Affective: Associated with interest, emotional reactions, identification, sense of belonging in the course, positive attitude about learning.
- Cognitive: Associated with students’ psychological investment in teaching and learning activities to master complex contents, use of learning or metacognitive strategies, learning goals, investment in learning.

However, emotional and cognitive engagement are considered less observable—and, thus, measurable—and gauged with internal indicators, including self-regulation, relevance of schoolwork to future endeavors, value of learning, personal goals, and autonomy as indicators of cognitive engagement, together with feelings of identification or belonging and relationships with teachers and peers [16]. In fact, Appleton et al. [16] admit that there are few instruments to reliably measure student engagement and, thus, they suggest the use of surveys and longitudinal studies such as the Student Engagement Instrument, the High School Survey of Student Engagement, or Kuh’s National Survey of Student Engagement [19], which are aimed to gather (rather than infer) the perspective of the student [20]. Although these strategies might be effective to measure student engagement in the medium/long term, they are unfeasible for measuring engagement in the short term (i.e., while students are attending the class) because they interrupt the learning process [21]. Therefore, instructors typically focus on the most easily observable dimensions of the engagement when delivering a training session: the academic and behavioral components [22,23], e.g., paying more attention to students that obtained a low score in a class quiz (i.e., academic engagement), or issuing challenging questions to those students that look distracted during class (i.e., behavioral engagement). Observed behavioral engagement is often considered an indicator for a certain degree of cognitive and emotional engagement [21].

Although the term *observable* has an implicit degree of subjectivity—which is again connected to instructor experience—instructors have been able to somehow *observe* students since the early stages of education. In fact, observing students—with the aim of inferring their engagement—in face-to-face education is straightforward due to the intrinsic nature of the learning environment. However, inferring student engagement in a virtual learning environment (VLE) poses several challenges [24,25]. Despite all the progress in terms of technology and methodology aimed at reducing the physical gap between students and instructors [26], VLEs still enable students to build a (virtual) wall between them and instructors, which prevents instructors from using the aforementioned traditional techniques—which have been shown to be effective in traditional face-to-face environments—to observe student interactions and reactions to infer their engagement [24]. Indeed, there are several factors that motivate this situation, for instance: instructors only see students’ faces in small windows, students can decide when their face is shown and voice is heard, or students can interact among each other without the instructor noticing, to name a few. In recent years, this situation has been further emphasized due to the consequences of the COVID-19 pandemic, where several instructors with little (or no) experience in distance education using VLEs have been forced to shift from face-to-face education to virtual education [27]. In this scenario, it has been acknowledged that the same techniques used to observe students in face-to-face learning to infer their academic and behavioral engagement are of little help in virtual education. In fact, instructors have realized that in a certain part of online synchronous lectures, the instructor cannot gather enough or any data to perceive the need to adjust training activities.

The purpose of this paper is manifold. First, it proposes a methodology to measure student engagement suitable for synchronous education in VLEs that does not depend on instructor experience. Second, using this methodology, it proposes a quantitative model to measure engagement in synchronous VLEs. More specifically, this model is built from the automatic measurement and systematic multimodal analysis of more than 30 types of digital features and events during the class. Third, to validate the feasibility of this approach, a software prototype has been implemented to measure student engagement in two different virtual environments: a masterclass and a hands-on session. These two scenarios present inherent characteristics such as different actor interactions (i.e., student–student, student–instructor, and instructor–student) and roles (i.e., active/passive) or classroom layout, which might impact student engagement. The obtained results aim to help those instructors who feel that the connection with their students has weakened due to the virtual nature of the learning environment.

This research takes advantage of the inherent digital nature of VLEs, in which automatically collecting and processing data by means of an automated software tool is more feasible than in face-to-face learning environments. Therefore, the contributions of this paper are the following:

**Theoretical Contribution 1.** A literature review of different strategies and tools to measure student engagement in synchronous training sessions carried out in VLEs.

**Theoretical Contribution 2.** A description of the methodological process to transform sensed data collected from synchronous lessons taught in VLE into educational insights (i.e., student engagement).

**Practical Contribution 1.** A proposal of an analytical model to measure student engagement obtained after instantiating the proposed methodology.

**Practical Contribution 2.** A proposal for the materialization of this model by means of a software prototype able to sense and process data in real time. This software aims to define the principles of a future-oriented tool for providing lecturers with information and valuable insights that have been traditionally easily available in face-to-face sessions but are currently unavailable in virtual classrooms.

The results of this research could be easily integrated with already existing tools for distance education that obtain alternative educational insights upon the analysis of the interactions between students and the learning management system [28].

The reminder of this paper is organized as follows. Section 2 reviews the existing approaches in the literature to measure student engagement in educational environments. Next, Section 3 details the selected methodology to identify a set of digital features that drive engagement in VLEs. Subsequently, Section 4 proposes a digital model that takes these features into consideration to measure student engagement in VLEs. Later, Section 5 shows a software prototype proposal implementation of the proposed model. Then, Section 6 describes the conducted experimental evaluation to assess the feasibility of the proposed approach. Next, Section 7 discusses the major findings of this research. Finally, Section 8 concludes the paper and enumerates possible future research directions.

## 2. Related Work

This section reviews (1) existing approaches in the literature to measure engagement in synchronous training lessons conveyed in VLE and (2) the available tools to assess the level of student engagement in VLEs.

### 2.1. Measuring Engagement in Synchronous Virtual Learning Environments

Face-to-face synchronous classes can be performed in bricks-and-mortar classrooms and virtual learning environments. An online classroom and a face-to-face classroom share many characteristics, but they also differ. In both environments, students are required to attend a class at a predetermined time. Although students in the face-to-face classroom are seated in front of the instructor, in the online classroom, their small bust images are on the instructor’s screen, very likely on multiple pages that need to be browsed manually. In the online implementation, students are free to turn off and on cameras and microphones, leaving and coming to the classroom without disturbing the attention of other participants. If in a face-to-face classroom, every participant’s movement can be a distraction for other participants, all this remains hidden from participants’ view and is often inaudible in an online classroom. In both environments, instructors must maximize instructional quality, answer class questions, motivate students to learn, and perform teacher-centered lectures that require passive learning by the students. All this in both environments is based primarily on vigilant student activities and behavior monitoring. Although it is an attempt to collect the same data, two completely different approaches exist. What can be done in a bricks-and-mortar classroom is not necessarily feasible in an online classroom.

Teaching activities need to be adapted according to the perceived engagement (e.g., behavior or response of students) in students [5]. This forces instructors to continuously collect real-time data about student activities and behavior. However, the obstacle to doing so is not always the instructor’s incapability to collect data but on processing these data. By the time teachers have a chance to slow down and reflect, they have already forgotten details of the observations or do not even remember what happened during the online lecture [5]. Nathan and Koedinger’s [29] concept of the “expert blind spot” is based on the cognition that “the more knowledge the teachers have, the harder it is for them to imagine the cause of the struggle that their students were facing”. This supports the idea of collecting and aggregating data on student behavior independently of the teachers’ actions to relieve and help them. Teachers can be partially relieved of personal observations by assigning learning tasks to the students on electronic devices (e.g., quiz, multiple choices) and later collect data about students’ work on a second-by-second basis at the expense of reducing personal communication with students. In addition, to maintain the benefits of relaxed learning in an environment without electronic devices, several attempts to capture data on students and their activities independent of electronic devices have been described in the literature. Ogan [5] describes this approach as instrumenting the physical space instead of instrumenting learners. Researchers experimented with cameras to detect facets [30,31], built toolkits for multimodal sensing of data about voice and facets [32], put cameras and other sensors around students’ necks to observe their motion and gaze [33], and instrumented classrooms with cameras and microphones to research the detection of behaviors in class [34]. These approaches are helpful in bricks-and-mortar classrooms but less so in synchronous online sessions.

The education shift from face-to-face to synchronous VLEs [27], motivated by the major change in 2020, stressed the difficulty of assessing student engagement in synchronous training sessions. Changed teaching circumstances require a rethinking of past developments in terms of finding use and upgrading, and developing where necessary. Due to teaching in the digital age, only the automatic measurement of engagement during virtual learning comes into play. Bosch [35] described methods for automated data collecting for student engagement assessment in VLEs. Among them, they suggested (1) the capture of various traits from image sensors (e.g., eye movement, facial expressions, and gestures and postures) and (2) tracing learner activities in online meetings (e.g., total time spent on lecture, number of forum posts, average time to solve a problem, number of submissions correct etc.). These methods extract features automatically and do not interrupt learners in the engagement-detection process. The methods in the automatic category are further divided into three groups:

- Log-file analysis: This consists of analyzing the context-dependent data associated with the registered traces and interactions (e.g., clickstream data) generated by the actors involved in the VLE. Typically, this analysis is conducted after the training activity when all the log files can be consolidated safely.
- Sensor data analysis: This consists of analyzing the data obtained from physiological devices (e.g., a fitness wristband) worn by the actors involved in the VLE.
- Computer vision-based methods: This consists of analyzing data (e.g., head poses) obtained from the webcam of each of the actors involved in the VLE by means of image-processing techniques.

In synchronous VLEs (e.g., online lecturers), it is possible to benefit from the last two. Captured data are known as external observable factors.

However, data alone do not yet provide helpful information to instructors. The use of methods based on computer vision and analysis of movements, postures, eye movements, and facial expressions is showing the most promising results in online lectures: a lot of research has been done to develop methods for inferring affective states based on data from sensors and computer vision [36,37,38,39,40]. Kapoor and Picard [38] described a three-stage process starting with sensing activity, followed by feature extraction (from face and head gestures, postures, and task information) and finished with affect classification. The difference from previous research was a novel fusion strategy. D’mello et al. [37] and D’mello and Graesser [36] developed and evaluated a multimodal affect detector that combines conversational cues, gross body language, and facial features. It uses feature-level fusion to combine the sensory channels and linear discriminant analyses to discriminate between naturally occurring experiences of boredom, engagement/flow, confusion, frustration, delight, and neutral. The analyses indicated that the accuracy of the multichannel model (face, dialogue, and posture) was statistically higher than the best single-channel model for the fixed but not spontaneous affect expressions. McDaniel et al. [39] focused only on the use of facial features to automatically detect boredom, confusion, delight, flow, frustration, and surprise. Correlational analyses indicated that specific facial features could segregate confusion, delight, and frustration from the baseline state of neutral, but boredom was indistinguishable from neutral. Dewan et al. [40] found that the most commonly used modalities in computer vision-based methods are facial expressions, gestures and postures, and eye movement. In fact, these computer vision-based methods are unobtrusive, cheap to implement and easy to use. Certainly, the procedure is very similar to a human’s (i.e., instructor’s) observations of student activities.

### 2.2. Tools for Assessing the Level of Student Engagement in Virtual Learning Environments

The use of video and audio recordings and data from sensors does not in itself contribute to the automation of the assessment of student engagement in the online and face-to-face class (also referred to as synchronous virtual learning). Instructors expect and need a plug-and-play tool. Otherwise, they will most likely insist on personally observing what is happening on the screen, or they will devote themselves entirely to the lecture while neglecting the importance of student engagement. As a tool, it is understood that an autonomous system program, which collects various data on facial features, sounds, events in the chat room, raising a virtual hand, and more, processes helpful information for the instructor and gives an overall assessment of student engagement. Before starting the development of a new tool, it is worth reviewing the existing comparable work.

Webb et al. [41] explored the use of Technology-Mediated Learning (TML) to support the development of the case method in face-to-face, hybrid, and entirely virtual (i.e., online) learning environments. Specifically, they suggest using web technologies to run asynchronous discussion boards (also referred to as forums) in the subject of Management Information Systems in a postgraduate degree. It is worth mentioning that class participation represented at least 50% of each student’s grade for this subject. In this work, authors obtained a notion of student engagement by analyzing the student–peer interactions in the discussion forums provided by the aforementioned technologies. In fact, authors perceived an increased level of student–peer interaction (i.e., engagement) thanks to the TML. Interestingly, authors also warn that, according to the research literature, attempts to introduce TML into the case method pedagogy may face serious resistance due to the reported preference of faculty members for traditional teaching over online teaching in a synchronous learning class.

The idea of student interaction with the web is further explored by Rodgers [42] in the context of an undergraduate module delivered using a mixture of traditional lectures and e-learning-based methods. All the e-learning-based materials were delivered via WebCT. In this context, the authors measured the level of engagement in the e-learning process as the number of hours spent online (i.e., hours logged into WebCT). The obtained results show that the coefficient of hours indicates that the level of engagement in e-learning has a positive, and statistically significant, impact on the module mark. Basu et al. [43] developed an Online Watershed Learning System (OWLS) over a Learning Enhanced Watershed Assessment System (LEWAS), which is a unique real-time high-frequency environmental monitoring system established to promote environmental monitoring education and research. It was inspired by Google Analytics to track users and their actions (i.e., mouse clicks, typed keys, and navigation through webpages) across devices in a cyberlearning system. This enables researchers to discover various engagement patterns/strategies taken by individual students to complete a given task. In this work, authors have used the time of participation on online platforms and the number of clicks as indicators of behavioral engagement in cyberlearning environments. Gangwani and Alfryan [44] did not propose any specific tool but, instead, they confirmed by means of surveys that there exists a significant positive correlation between online teaching strategies: instructor strategy, student interaction strategy, strategy, student motivation strategy, institutional strategy and overall student engagement.

Similarly, MacRae et al. [45] did not propose any specific tool to quantify the level of engagement, but they assessed it from the analysis of the written feedback (i.e., comments) from students. Garcia-Vedrenne et al. [46] described the experience of migrating a flipped classroom to a remote learning environment. In this scenario, they used participation and attendance to quantify the level of student engagement. In this regard, they used Google Forms to track attendance and collect real-time student responses to questions posed during class and log attendance, and interactive participation tools in Zoom, including polls and chat, to engage students directly during the lecture. Interestingly, the authors confirmed that in this experience, those students who were able to use both audio and video were often more engaged than those that chose who did not or who were unable to do so. Caton et al. [47] assessed student engagement by analyzing student question-asking behavior, i.e., the amount and type (i.e., confirmation or transformation) of questions that students ask during a session. Heilporn and Lakhal [17] confirmed that assessing how student engagement is developed in HyFlex [48,49] courses (i.e., a combination of Hybrid and Flexible: one the one hand traditional and blended learning are used and, on the other hand, students can chose their mode of attendance) is still vague, since there is little knowledge about instructional strategies fostering student engagement in HyFlex courses. To assess the student engagement in a HyFlex course, students were asked in open-ended and close-ended questionnaires to link the main components of the new course design with indicators of student engagement. Ayouni et al. [50] discussed how the construct of student engagement in an online setting refers to the fact that the more students learn about a subject, the more likely they are to know about it, and the more feedback they get on their work, the deeper they come to understand what they are learning and the more they become collaborative. Nonetheless, the authors confirmed that despite the interest in student engagement in online learning settings, most studies either reduce this construct to the number of hours spent online or adopt the same conceptualization given in traditional learning settings. Therefore, they proposed to capture the dynamic nature of the behavioral, social, cognitive and emotional dimensions comprising student engagement in an online context. In this regard, they proposed more than 30 parameters to be extracted from the LMS to quantify student engagement, such as number of discussions and replies posted, forum participation, or number of emails sent to instructor, among others.

Overall, the following tools have been extracted from the literature review to quantify the level of student engagement in a VLE:

- Online interactions in web technologies [41] using user-tracking systems such as Google Forms and interactive participation tools in Zoom; [46], Google Analytics or Learning Enhanced Watershed Assessment System (LEWAS) [43];
- Hours spent in web technologies [42];
- Surveys and longitudinal studies [16,17,20,44] such as CLASSE, the Online Student Engagement Scale, the Student Course Engagement Questionnaire, the University Student Engagement Inventory, or the National Survey of Student Engagement [19].
- Student question-asking behavior [47].

This work proposes an alternative approach aimed at providing instructors with an autonomous software tool that gives them a global view, in real time, of the overall student engagement by systematically analyzing the digital parameters that can be distilled from the VLE. This tool will relieve instructors of the arduous task of real-time engagement monitoring in synchronous VLEs and allow them to continuously adapt their sessions to keep the engagement in students at the desired levels. The following section describes the methodological process to identify the parameters that drive engagement in synchronous VLEs.

## 3. Methodology to Model Engagement from Sensor Data in VLEs

**Figure 1:**
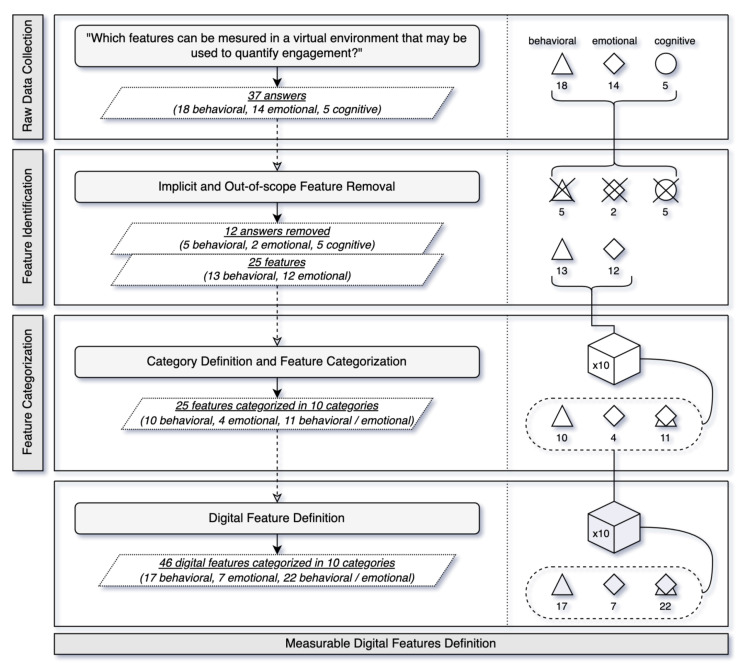
Methodological approach to model engagement in virtual learning environments.

With the aim of determining a model to quantify student engagement in synchronous VLEs, a methodology that is explicitly focused on defining a set of standardized manageable and measurable digital features has been designed and implemented (see Figure 1). This set of measurable digital features constitutes the set of inputs of the quantifiable model for measuring the engagement from the (digital) data sensed in synchronous virtual lessons.

As shown in Figure 1, this four-stage methodology starts off with an initial collection of features (i.e., raw data collection), an identification of features and their categorization until it finally obtains a set of measurable digital features. The stages and their corresponding responsibilities are as follows:

1. **Raw Data Collection**. Refers to the collection and typification (in behavioral, emotional and cognitive terms) of the responses from experts in education to how engagement can be measured in a synchronous virtual learning environment.
2. **Feature Identification**. Transforms the raw data into measurable features. It also discards those features that are either implicit in other features or are out of the scope of the synchronous virtual scenario.
3. **Feature Categorization**. Classifies the (refined) features obtained in the previous phase through definition and corresponding grouping in a range of manageable categories. This operation contributes to enhancing both the degree of understanding and the usability of the engagement model, as well as improving its level of customization.
4. **Measurable Digital Features Definition**. Defines the final set of measurable digital features that will be used to quantify student engagement in synchronous virtual sessions. A quantifiable unit and a level, albeit it individual (student) or group (class) are established for each digital feature.

This process and the results obtained in the execution of the methodology are further described below. Please note that for the sake of this research, all the stages were conducted in the context of the HOlistic online Teaching SUPport (HOTSUP) Erasmus+ project (Reference number: 2020-1-PL01-KA226-HE-096456) that started in April 2021. More specifically, the interdisciplinary panel of experts that executed all the stages of this methodology was composed of: three instructors from the Computer Engineering Department at La Salle Campus Barcelona (Universitat Ramon Llull, Spain), three instructors from the Faculty of Logistics at University of Maribor (Slovenia), three instructors from the Human Sciences Department at Libera Università Maria Ss. Assunta (Italy), three instructors from the Faculty of Management Engineering at Poznan University of Technology (Poland), and two teaching experts from ValueDo (Italy).

### 3.1. Raw Data Collection

In this first phase, all the participants from the higher-education institutions involved in the consortium of the HOTSUP Erasmus+ project were asked via email to answer the question “*Which features of a synchronous virtual lesson can be measured and therefore used to quantify student engagement?*”. The responses obtained were the starting point for the identification of features in subsequent phases.

A Miro board [51] was selected to collect the responses. This web-based platform offers the creation of common workspaces through virtual whiteboards, where multiple users can collaborate in real time with ease. In this case, a panel was configured with three different areas that correspond to the types of engagement (behavioral, emotional and cognitive) and a brief description of each. These types of engagement were chosen based on widely accepted definitions of engagement [16,17,18]. It is worth noting that the academic dimension of engagement has been excluded due to the unfeasibility of being quantified by means of audiovisual data during a synchronous virtual lesson. Additionally, a set of color-coded notes (“Post-It” style) was configured and each of the participating institutions was assigned a color. This was aimed at easing participants into responding to the question and the analysis of their contributions.

A total of 37 features were uploaded. A breakdown of the responses can be seen in Table 1, Table 2 and Table 3. In these tables, the “ID” column represents the identifier of each feature, and the “Feature” column includes the actual responses.

More specifically, Table 1 includes the 18 behavioral-type answers obtained, which represent 49% of the total answers collected. As can be seen, data on multiple aspects of interaction (lecturer-student, student–student), participation (i.e., raised hands, posts, screen sharing, voice interventions) and attendance were collected. Table 2 shows the 14 types of emotional features identified, which make up 38% of the total collected answers. Many of these behaviors stem from the emotional state of the student at the time (i.e., type of stickers posted in chat, yawning, students looking around). Finally, Table 3 shows the five answers that correspond to cognitive engagement, which accounts for 13%of the total answers collected. Answers in this category mostly deal with the amount of time required to master the subject (i.e., time to persevere, number of correct answers).

Surprisingly, data collected at this stage are correlated with the ontology proposed by Ayouni et al. [50], which endorses the validity of the results obtained at this stage. However, it is worth noting that data from [50] refer to features collected upon the interactions between students and the learning management system, while data collected in this study refer to features collected upon the interactions between students and the VLE.

### 3.2. Feature Identification

Once the answers for the previous phase had been obtained, the set was then further refined. For this part of the study, 37 responses were analyzed to identify and discard those features that could be considered either implicit (i.e., already included in other more specific features) or unfeasible to measure/quantify and, thus, out of the scope. As can be seen below, the answers classified (and discarded) as out of scope are those that require additional information beyond what can be sensed from the student interactions with a VLE while attending a synchronous training session (where sensed data can only come from the audio, video, and chat). Thus, we leave aside interactions with other platforms such as the learning management system.

Table 4 shows the 12 rejected answers that were consequently eliminated by the panel of experts from the set of 37 features obtained in the previous phase. The “ID” column indicates the single identifier, and the “Answer” indicates the feature, both of which correspond to the respective content of Table 1, Table 2 and Table 3. The “Reason” column denotes the reason for rejection. As can be observed, three features (i.e., B1, B2, and B9) were eliminated as they were considered to be overly general and already included (implicit) in other more specific elements. For instance, the degree of student interaction and participation can be obtained through other features such as B4 (total duration of the microphone on for each participant), B5 (number of raised hands), B6 (number of posts in meeting chat), and B7 (number of screen sharing). B3, B12, C1, C2, C3, C4 and C5 were discarded because they require integration with other systems external to the videoconferencing software, so they require additional data beyond audio, video, and chat. In addition, E4 and E9, although capturable by video signals, were also discarded as they were considered to be unfeasible to measure/quantify given the typical framing (upper torso of the body) of the camera.

Taking into consideration the previous decisions, this phase concluded with a total of 25 features (see Table 5) which were used in the next phase (Feature Categorization). The results obtained were considered suitable to quantify engagement in synchronous virtual learning sessions, given that they describe events that are strictly measurable from data naturally generated in these environments.

### 3.3. Feature Categorization

To obtain a greater degree of understanding and manageability, this stage included the classification of the 25 features extracted in the previous phase (Feature Identification) according to the digital aspect to which they refer.

Specifically, the grouping resulted in a total of 10 digital categories, which contained the 25 aforementioned features. Table 6 provides a breakdown of the results. The column “Digital Category” includes the name of the digital aspect, “Engagement” includes the typology or typologies of engagement covered by the category, “No./% total” details the number of features grouped in this category and their percentage with respect to the total (of 25 features), while the last column “Features” indicates the features classified in the corresponding categories preceded by their unique identifier. As can be seen, four digital categories have been defined to group the identified features related to behavioral engagement (i.e., voice interactions, hand raising, screen sharing, and sound analysis), two digital categories have been defined to group the identified features related to emotional engagement group behavioral features (i.e., facial emotion and eye gaze), and four digital categories have been defined to group the identified features related to behavioral and emotional engagement (i.e., attendance, camera usage, chat interactions, and mouth-movement analysis). it should be noted that these last four digital categories include both behavioral and emotional features, while “behavioral” and “emotional” exclusively include features of the respective types. This combined type of engagement is not surprising, since a digital aspect, such as “camera usage”, can include both behavioral and emotional features, as in this case when B17 and E13 are included, i.e., the modeling of the use of the camera in this case includes whether or not the student has activated their camera (derived from behavioral engagement) and/or switched it off (typical of emotional engagement), which explains the two types of engagement present in this relationship.

As the next section shows, this classification offers both a greater understanding of the digital features defined, as well as a convenient way to configure a custom engagement measurement model according to the teaching activity that takes place in the synchronous virtual session.

### 3.4. Measurable Digital Features Definition

Taking as a starting point the 10 previous categories and their respective 25 features, the objective in this phase was to specify a sufficient set of measurable digital features. This definition comprised (1) the identification and quantification of features to be sensed/detected/calculated and (2) the choice of level (individual/student or group/class) to which they refer. This section specifies how each one of the defined digital categories can be objectively quantified.

Table 7, Table 8, Table 9, Table 10, Table 11, Table 12, Table 13, Table 14, Table 15 and Table 16 show the details of this definition for each of the 10 digital categories previously presented in Table 6. The “Level” column indicates the level (i.e., group or individual) and the “Measurable Digital Feature” column indicates the digital features defined.

Table 7 shows how the category “attendance”, and its respective two features (i.e., B17 i E13), were specified in four digital features, i.e., three at the group level and three at the individual level. At the group level (i.e., ATG1, ATG2 and ATG3) they provide enough detail to quantify both current attendance and the attendance extremes (maximum and minimum) throughout the session. Furthermore, the aforementioned table also takes into account the duration of each student’s attendance for the session (i.e., ATI1).

For the “camera usage” category, Table 8 details the resulting digital features defined for the two features, namely B15 and E7, identified in the previous phase. It includes features both at the group level (i.e., CUG1, CUG2 and CUG3), and individual (i.e., CUI2, CUI3 and CUI4), referring to the state (on/off) of the camera as well as its deactivation. At the individual level for each student, the current state of the camera (CUI1) is also defined. In this way, seven measurable digital features are defined from the two features grouped in the category.

For the “voice interactions” category, Table 9 details the seven digital features that were defined for the four features identified in the previous stage (i.e., B4, B11, B13 and B18). In this case, four features are described at a group level (i.e., VIG1, VIG2, VIG3 and VIG4) which focused on the length of the interventions of the participants in the session (professor and students), the number of interventions of the students and the instances of silence. On an individual level, three features (i.e., VII1, VII2 and VII3) that describe the interventions of each of the students are included.

Table 10 shows the results of the “hand raising” category. The B5 feature identified in the previous phase encompassed three measurable digital features, one of which was for group (i.e., HRG1) and the remaining two for individuals (i.e., HRI1). Similarly, Table 11 displays the results for the “screen sharing” category. From the B7 feature identified above, one feature was included at the group level (i.e., SSG1) and two at the individual level (i.e., SSI1 and SSI2), regarding the screen-sharing functionality.

Regarding the “chat interactions” category, Table 12 shows the details of the definition of measurable digital features extracted from the features B6, B14, B16 and E1 of the previous stage. At the group level, three digital features were included and at group level 4. As can be seen, the defined digital features provide significant findings on the use of chat, not only on the number of messages at the group (i.e., CIG1 and CIG2) and individual (i.e., CII1 and CII2) levels, but also information of interest such as the type of stickers used (i.e., CIG3 and CII4) and how many of the messages were in the form of a question (i.e., CII3).

As for the “facial emotion” category, Table 13 highlights the two digital features that were defined from the E10 feature of the anterior phase. Both at the group level (i.e., FEG1), and at the individual level (i.e., FEI1), the detection of the students’ strongest emotions is included.

For the “sound analysis” mapping, Table 14 shows the four digital features defined at the group level (i.e., SAG1, SAG2, SAG3 and SAG4), which directly correspond to the four features E3, E11, E12 and E14.

Table 15 shows the measurable digital features derived from B10, E2, and E6 in the category “mouth-movement analysis”. In this case, the quantification of yawning as a sign of boredom is included, both at the group level (i.e., MMG1) and individually (i.e., MMI1), as well as the interaction with people outside the virtual context of the session, also at the group level (i.e., MMG2) and individual level (i.e., MMI2).

Finally, Table 16 shows the results for the “eye gaze” category, and its three emotional features (i.e., E6, E5 and E8) identified in the previous phase. Specifically, five digital features were defined, two at the group level and three at the individual level. In this case, both at the group and individual level, the action of moving away from the framing area of the recording (i.e., EGG1 and EGI1) and not looking at the screen (i.e., EGG2 and EGI2) are defined. At the individual level, the percentage of attention of students in English is also defined (i.e., EGI3).

The above definition has taken into account the 10 categories defined in “feature categorization”, and their corresponding 25 features, to obtain a total of 46 digital features (i.e., parameters that can be sensed in a synchronous virtual lesson). Table 17 shows the number/percentage of initial features (i.e., column “No/% initial features”), and the number/percentage of digital features (i.e., column “No/% digital features”) defined for each of the categories.

To sum up, the final collection of measurable digital features (46 items) is larger than the initial set (25 items), since for almost all the categories (9 out of 10) multiple digital features are proposed to measure each one of the categories identified at the first stage of the process. Indeed, these digital features model actions and events linked to the behavioral and emotional engagement, respectively. This set of parameters will be used as input in the calculation of the quantifiable model for measuring engagement from digital features, which is explained in the next section.

## 4. A Quantifiable Model for Measuring Engagement from Digital Data

This section proposes a model aimed at quantifying the overall engagement of a class, taking into account the set of digital features identified thanks to the methodology described in the previous section. It is worth mentioning that this model should also include options for its customization, being adaptable to the intrinsic needs of the teaching activity that takes place in the teaching–learning process, as well as to the preferences of the teaching team.

For the definition of the proposed engagement quantification model, four main premises have been established:

1. **The level of engagement detected must be self-explainable.** In this way, the model is presented as easy to understand and useful and as an additional complement to the lecturer’s perception and experience [2,6].
2. **The behaviors and emotions of the individual students detected, as well as their respective changes, must imply variations in the level of engagement.** This gives the model coherence with respect to what actually happens and is sensed during the virtual session, so that the lecturer can observe in the model the student engagement evolution during the teaching activity.
3. **The quantification of engagement must be configurable according to the teaching activity.** Just as the model provides information that complements the lecturer’s skills, the model should also be able to be fine-tuned according to the lecturer’s preferences, thus offering the possibility of providing adaptability to the intrinsic characteristics of the teaching scenario that takes place.
4. **The level of engagement should tend to decrease over time and should be adaptable according to the teacher’s preferences.** According to the literature, it has been found that student concentration, attention to, and retention of lecture materials decline and decay according to the time spent on task [53,54], which unavoidably should impact student engagement.

Regarding the first premise, to ease its explainability, the representation of engagement has been established by means of an integer number, with a range of possible values from 0 to 100, which has been labeled ENQUA (ENgagement QUAntification). Thus, from the lowest to the highest possible values of engagement, the value 0 indicates that no sign of engagement has been detected by the students, and the value 100 indicates that the group of students shows behaviors and emotions that are interpreted as total engagement in the teaching activity.

In reference to the second premise, the effect of behaviors and emotions (as well as their variations) on the level of engagement, the 10 categories and their corresponding digital features obtained in the last phase of the methodology presented in Section 3 have been taken into account according to Equation (1):

(1) $$SensedData=\sum_{\forall i}DC_{i}*W_{i}$$

where:

- $DC_{i}$ is each one of the 10 measurable digital categories proposed in Section 3 quantified in the range [0,1]
- $W_{i}$ is a parameter in the range of [0,1] corresponding to the contribution of the *i-eth* digital category to the ENQUA.

In this way, it is possible to take into consideration the different behaviors and emotions detected in the group of students to model what is actually happening in the virtual classroom and which translates into variations in the ENQUA. As for their weighting in the calculation, a weighting has been established at the category level, and not at the level of digital features, so that the digital features of each category contribute equally. The reason for this decision is two-fold: a weighting at the digital feature level could (1) mask increases or decreases in ENQUA and (2) compromise the degree of ease of use for the teacher, since it would entail the definition of an unmanageable set of parameters.

Regarding the third aspect, the ability to customize the model according to the teaching activity, three considerations were made: (1) the definition of a contrasted set of teaching activities, (2) the definition of a scale of values to establish different degrees of influence of the categories, and (3) the selection of a set of default weight values according to the teaching activity as a proposal and with the possibility of manual refinement by the teacher.

In reference to the set of teaching activities defined and the associated teaching activity typology:

1. **Lecture.** Based on the presentation of concepts and/or exemplification of their use, usually including student participation in the form of questions.
2. **Tutorial/Hands-on.** Focused on problem solving without the use of laboratory material.
3. **Laboratory class.** Focused on problem solving with the use of specific laboratory material.
4. **Seminar.** Include problem solving with the use of specific laboratory material.
5. **Doubts session.** Specifically oriented to the resolution of student doubts.
6. **Peer instruction.** Students exchange knowledge about aspects proposed by the teacher.

In reference to the scale of values to weight each of the categories, and thus be able to configure their influence in the calculation of engagement, five labels and values have been defined with respect to 100% of engagement. Specifically, these are: irrelevant (0%), slightly relevant (25%), relevant (50%), fairly relevant (75%) and very relevant (100%). In this way, and with the defined teaching activities and the scale of influence values, a set of default values has been determined for each of the 10 categories, and consequently, for the corresponding digital features. Table 18 shows the details of the obtained results. In the first column there are the 10 digital categories identified in Section 3 and in the heading of the following columns there are the six teaching activities, so that for each teaching activity the proposed default influence values are indicated. These values are represented according to the acronyms: irrelevant (I), slightly relevant (SR), relevant (R), fairly relevant (FR) and very relevant (VR).

Finally, the overall engagement over time (i.e., ENQUA(t)) for a group of students attending a synchronous virtual lesson should include (1) the decreasing nature of student engagement over time [55] and (2) the weighted sum—as similarly done by Alves Durães [28]—of the digital events identified in the 10 digital categories propeosd in Section 4, and also be adaptable to lecturer preferences. In this regard, we propose the following analytical model:

(2) $$ENQUA\left(t\right)=\left[\alpha *(e^{-t*K})\right]+\left[\beta *(\sum_{\forall i}DC_{i}*W_{i})\right],$$

where:

- α is an instructor-defined parameter in the range of [0,1] that determines the influence of the decreasing nature of student engagement over time.
- *K* is an instructor-defined parameter in the range of [0,∞) that quantifies the natural capacity of students to disengage from the class over time. Small values of *K* mean that it takes large amounts of time for students to lose engagement.
- β is an instructor-defined parameter in the range of [0,1] that quantifies the prevalence of the behaviors and emotions (selected digital features) detected. It must be computed as (1−α).

These design decisions have made it possible to obtain a model aimed at quantifying student engagement from the sensed data in a VLE, including mechanisms that simulate (its natural tendency to) decrease over time. Additionally, the model offers mechanisms that give it a high degree of customization and adaptability to the needs of teaching practice (weighting of the categories of digital features according to the teaching activity) and teacher preferences (for both, the capacity of students to disengage from the class, and the influence of the decreasing nature of student engagement over time and the prevalence of the behaviors and emotions detected).

## 5. Conceptualization of a System Prototype

This section discusses a possible implementation and its technological feasibility of a software prototype for the proposed engagement quantification model. To begin with, and as elements to be taken into consideration for the conceptualization of the system, the authors believe that ease of use and security (i.e., sensed data are very sensible) are paramount. The system should be easy to use, meaning that it should minimize the use of additional technology and/or devices other than those commonly used in a synchronous virtual learning environment, such as computers, video cameras and Internet connections. In addition, it should offer a high degree of usability, understood as being able to quickly find the query about the engagement (and the information derived from it) detected in the session. In terms of security, the system must minimize the risk of improper access to data, thus providing adequate mechanisms to protect the information derived from the monitoring of the set of digital features.

Considering the above premises, a client–server architecture has been selected as an appropriate basis for the conceptualization of the system. Specifically, the following roles have been defined for each of the components:

- Server. Acts as another participant of the virtual classroom that collects and processes all environmental data (video quality, audio quality, and facial landmarks for engagement measurement) emitted during the lesson to implement the ENQUA(t) function. Additionally, it hosts a web dashboard to visualize all the information regarding the engagement.
- Client. Allows, to authorized users, the visualization of the data collected and processed by the “server” through a web browser. The presentation of the information takes place through a web dashboard, hosted by the server, that offers several sections in reference to the measured engagement.

The choice of this architecture and the associated role definition offers capabilities to provide the right answers for both the ease of use and the implementation of security measures (both for data storage and data transmission). Regarding the first one, several web dashboard visualization models are possible, since only one device (i.e., computer desktop, laptop or mobile phone) with a web browser installed is required. In this regard, access is possible both from the same computer that acts as “server”, as well as from another device, including (without any additional configuration) simultaneous viewing from multiple devices. This capability, and its corresponding visualization modes, also entails another functionality that contributes effectively to increase the usability of the system. Thanks to it, the teacher can access the web dashboard through the same device as that from which he/she connects to the virtual learning environment (e.g., Zoom, Teams, etc.), or he/she can use an additional device. In the latter case, it is possible to have the web dashboard on another screen, avoiding a possible problem of organization and/or of the available usable space on the screen where the connection to the VLE takes place. This facilitates operations such as the display of the video-conference program itself, screen sharing and/or the execution of multiple programs. Additionally, in terms of ease of use, the web dashboard can use graphics to facilitate a quick interpretation regarding the engagement status.

In reference to security measures, the storage of information can be done on the lecturer’s computer (when acting as a “server”), or on another device on the network (when “client” and “server” are different computers). The first model avoids extra data traffic over the network, and in the second, it is necessary to establish a secure communication protocol such as hypertext transfer protocol secure (HTTPS). In this sense, it should be noted that for a higher degree of security for both data storage and transmission, the “server” can be in a “known” computer in a local network duly protected rather than in an “unknown” cloud server. In addition, the “server” can generate an auto-generated and specific password for the session being monitored, so only authorized persons have access to the protected web dashboard that allows viewing the engagement information.

**Figure 2:**
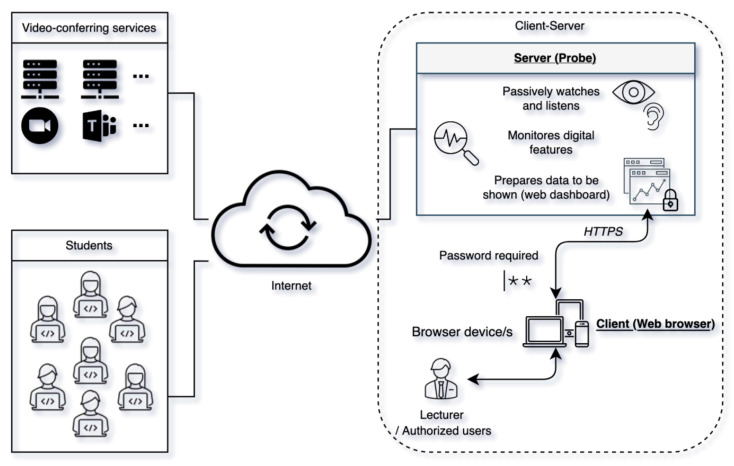
System prototype conceptualization, components and interactions.

Figure 2 illustrates the system characteristics explained up to this point, corresponding to the box on the right labeled as “client–server”, and establishes the necessary relationships among all the components and systems involved for their interconnection. “Video-conferring services” represents the videoconferencing tools (e.g., Zoom, Microsoft Teams, ⋯) that provide the virtual learning environment. “Students” models the group of students connected to the environment where the virtual session takes place. “Client–server” groups the “client” and “server” components of the conceptualized prototype, “server” monitors the session and provides a password-protected website that allows access to the engagement information. “Client” is the device, equipped with a web browser, used by the teacher and/or authorized users (by password) to view the web dashboard generated by the “server”.

With regard to the implementation of the software component that monitors the session, this can be carried out using a strategy focused on image capture (i.e., processing screenshots) or based on the analysis of the HTML code of the VLE’s web platform using a web driver such as Selenium [56].

The aforementioned decisions offer different ways to design an execution flow, from the start of the monitoring system to its completion. A possible scheme is as follows: (1) configuration, (2) access details, (3) engagement monitoring, (4) authentication, (5) visualization and (6) session ending. First, by interacting with the “server”, it is possible to specify the characteristics of the session to be monitored, so the system then generates the access credentials and the URL of the web dashboard for password-protected visualization. From this point the “server” would start monitoring the system while keeping the web dashboard updated. The visualization, which can be done using the different modes explained above, is performed by the “client”. To do so, the teacher authenticates at the indicated URL to be able to visualize and interact with the web dashboard. Finally, and from the “client” program, the teacher can order the system to stop when he/she deems it convenient.

## 6. Experimental Evaluation

To evaluate the potential of the presented system conceptualization, an initial prototype has been developed and tested. For this purpose, two different learning activities have been chosen: a masterclass and a hands-on class. These activities, corresponding to “lecture” and “tutorial/hands-on” typologies presented in Section 4, respectively, have been selected to test the prototype functioning because of their dissimilarities, which should show significantly different results and allow a comparison in accordance with their intrinsic characteristics.

It is worth mentioning that the hands-on class consisted of two distinct parts: (1) an initial block, of short duration, focused on the acquisition of specific knowledge in the field of web development and (2) a part for the illustration and implementation of the initial knowledge. Interaction among students is not as common in the first part as in the practical part, since it is during its application that doubts normally arise regarding the application of concepts in a specific scenario proposed by the teacher.

**Figure 3:**
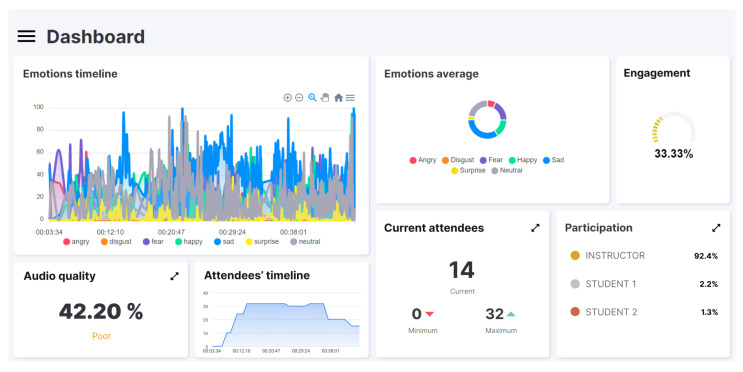
Software prototype, masterclass results.

**Figure 4:**
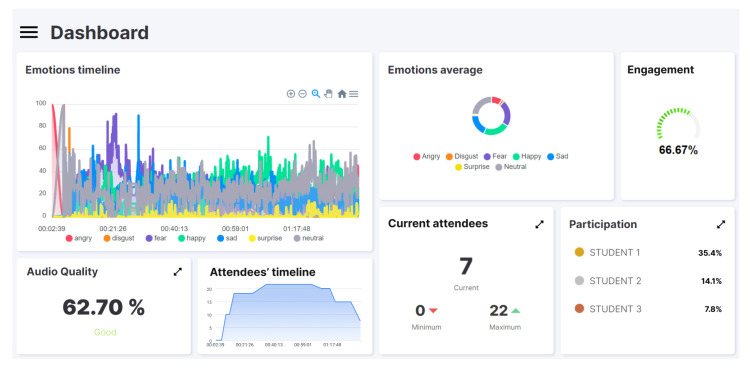
Software prototype, hands-on results.

Figure 3 and Figure 4 correspond to the screenshots of the web dashboard for the two tests performed using the software prototype: the masterclass and the hands-on, respectively. Both show information regarding engagement (“engagement”), emotions (“emotions timeline” and “emotional average”), audio quality (“audio quality”), attendees (“attendees’ timeline” and “current attendees”) and participation (“participation”). These data provide information that complements what the lecturer perceives, and it can be affirmed that they provide a similar vision (or even more detailed) to what he/she could perceive by him/herself in a face-to-face environment. Specifically, it is possible to observe (1) the predominant emotions of the students as well as their variations, (2) the distribution of the predominant emotions, (3) the level of engagement calculated at each moment, (4) the quality of the audio, (5) the evolution of the number of attendees and their minimum, maximum and current values, and (6) the level of participation and its distribution. The following characteristics can be observed.

In reference to the calculated engagement metric, a clear difference can be observed between the two types of class. The masterclass session has an engagement rate of 33%. In contrast, the hands-on session triples this value to 66%. Regarding participation, and with a manifest correlation with the engagement metric, in the masterclass the instructor is the protagonist (i.e., the one with highest participation), opposite to what it can be seen in the hands-on session where the students are the ones who participate the most. Specifically, in Figure 3, the instructor invests 92.4% of the time explaining concepts, as opposed to the 3% belonging to the student who has participated the most. Conversely, in Figure 4 it can be seen that students accumulate more than 55% of the time of the session. Regarding emotions, two observations are noteworthy. First, comparing the “emotions average” charts, it can be seen that the predominant emotion detected in the masterclass is “sad” (sadness), compared to “happy” (happiness) in the hands-on class. From the “emotions timeline” graphs, changes can be observed in the mood of students when the realization of an experiment is communicated at the beginning of the class (to meet ethical regulations), as well as a change when the theoretical part ends and the practical part of the hands-on session begins (minute 21).

The results obtained using the implemented initial prototype have allowed the validation of the potential of the proposed model and the suitability of the conceptualization of the system for the purpose presented in this work.

## 7. Discussion

The designed model is explicitly oriented to the teaching staff, presenting itself as a support tool oriented to perform an adaptive teaching practice according to the level of engagement induced by the interactions of the participants with VLEs. The model is not oriented to the evaluation of students and their behaviors; the interpretation of the results must be directed to assess the adequacy (and adaptation of the variations) of the teaching practice according to the level of engagement of the students. The knowledge offered by the model, used as a complement to the lecturers’ knowledge and experience, is a guide to deploy adaptive teaching practices that show a high potential to contribute to design proper environments oriented to favor the achievement of competencies and learning outcomes. With the observed potential, both actors (lecturers and students) involved in the teaching–learning process can benefit from a more effective and efficient teaching practice. On the one hand, lecturers can take advantage of objective and key information about the state of the group of students that can be used for guided decision making; on the other hand, students obtain a scenario adapted to the needs of the teaching practice.

The rationale behind this research is the reduced number of alternatives to assess student engagement synchronous virtual sessions. Some instructors have realized that they do not know what exactly is going on in the virtual class and, also, they often lose contact with the participants; thus, they are not sure how many participants are effectively following the development of the lesson. In fact, it is difficult to observe what is happening with participants of the online event on the screen while, at the same time, lecturing the learning content synchronously. This may lead to situations in which the importance of student engagement is neglected by instructors in favor of communicating the learning materials. For instance, as can be seen in the masterclass example (see Section 6), the instructor lectured without encouraging students to change emotions from “sad” to “happy” or at least to “neutral”. Indeed, manually monitoring each participant in the synchronous virtual learning environment is exhausting and distracting. Therefore, checking the proposed dashboard (see Figure 4) seems very easy and intuitive, since it only requires a quick look from the instructor a few times during the synchronous virtual learning event. Therefore, although the proposed tool is helpful in all forms of synchronous virtual learning environments, we believe that its benefits are emphasized in those teaching scenarios where the instructor’s activity predominates and the students are in the role of passive listeners—which may favor potential disengagement.

The proposed model is suitable for any kind of training conducted in a synchronous virtual learning environment. We believe that it might be especially attractive for secondary education (perhaps less for tertiary education), where students are more prone to disengagement. Furthermore, in those situations where there is a transition from a face-to-face environment with small groups to a synchronous virtual learning environment with larger groups, students might be more tempted to disengage from the learning process [57]. This research aims to fight the drop in student engagement experienced in recent years by several educational centers due to the shift from face-to-face to distance education. So far, several instructors are eager to return to a face-to-face environment because they feel lonely “on their side of the screen”. We believe that practical use of the proposed tool would convince a certain part of these instructors to use it and feel more comfortable in synchronous virtual education. We see the tool as a convenient aid for instructors, regardless of their teaching experience. For instance, instructors were confronted with a virtual learning environment in 2020 for the first time and most of them are still experimenting with how to effectively carry out pedagogical activities. Monitoring participant engagement can make the transition faster and easier for both participants and instructors.

We anticipate that the frequency with which instructors use the tool may impact the overall learning process performance. The tool could be used for either an extended period of time (i.e., all sessions) or only a few lessons (e.g., first meetings with the new group, at the beginning of their instructor journey, in really important lectures that are the foundation for understanding everything else). It may also be interesting when used by those instructors that feel themselves underestimated by students, so that in this way, they could try to identify the possible causes of poor engagement among participants. These are discussed below.

The proposed methodology has made it possible to obtain an exhaustive set of measurable digital features in synchronous VLEs. Based on this characterization, and with established design premises to obtain an objective assessment of student engagement, a model for its quantification has been defined. For its materialization, a conceptualization of a system has been designed, which has led to the development and testing of a prototype. The results obtained have allowed the affirmation of both the potential of the model and its technological feasibility. The authors observe certain limitations related to its scope of application, to the system’s conceptualization functionalities, and others related to the maturity of its implementation.

Regarding the scope of application, the model does not include all the dimensions of engagement, since, with limited data collection to those that can be captured by devices used in standard videoconferences (camera, microphone and chat), it is focused on behavioral and emotional dimensions. In this way, the model does not use data on academic performance, which impacts academic and cognitive dimensions of engagement. This limits the applicability of the proposed model to be used in synchronous VLEs where information related to psychological investment is not required (i.e., time on task, homework completion, credits earned). For those situations where this information is needed, these data could be obtained, for example, through interaction with LMS modules (i.e., log-file analysis).

Concerning the functionalities, both the system’s conceptualization and the developed prototype offer crucial and useful information about the level of student engagement in synchronous VLEs. The potential of this knowledge lies in its use by the lecturer, which is a valuable guide that can complement his/her experience, offering an objective basis for, for example, deciding to change the teaching activity. However, the system does not include dynamic suggestions based on the quantification of engagement, as an intelligent recommender system could do based on previous experiences. To address this limitation, a possible extension would be to provide the system with mechanisms to store relationships between the teaching methodology and the level of engagement detected, to show (to the lecturer) recommendations based on a knowledge model obtained from a system of association rules or case-based reasoning.

In reference to the maturity of the conceptualized system, the implementation of the prototype has enabled the positive assessment of both the potential of the model and its technological feasibility. The results obtained are encouraging and allow the observation of its usefulness in contributing to the design of teaching environments propitious to the achievement of learning objectives. However, further development steps need to be conducted to make the software tool easily distributable and installable—so far, there exist several library and environment dependencies that need to be wrapped into a single software package.

## 8. Conclusions and Future Work

The methodological approach to model engagement presented in this work has led to the obtaining of a set of 10 categories that group a total of 46 digital features measurable in virtual learning environments. These features have been directly obtained from experts in education to how engagement can be measured in a synchronous virtual learning environment.

It is worth noting that the purpose of this research is not to assess students in terms of behavior to provide some insight when grading but to provide instructors with an additional tool to relieve them from real-time engagement monitoring in VLEs and allow them to continuously adapt their sessions to keep the engagement in students at the desired levels.

The designed model provides objective information on the level of student engagement, which is a powerful aid (for the lecturer) on the decision-making process during session development to maintain the desired level of engagement. The decisions made by the lecturer can lead to observable changes thanks to the model’s design premises, which effectively contributes to the continuous improvement of the teaching practice. Based on work by Czerkawski and Lyman [58], focused on how to incorporate best practices for student engagement in online learning, the authors identify and derive aspects that they believe applicable and noteworthy to take into consideration to improve the level of engagement in synchronous education in VLEs. Interaction and collaboration between lecturers and students are presented as key elements that determine the level of engagement.

Dixson [59] states that it is not the type of teaching activity (active or passive) that affects the level of engagement, but the level of interaction during the activity. In relation to this, the role of the lecturer can be seen not only as an expert in the area of study, but also as an advanced learner and mentor capable of designing teaching activities in which participation plays a prominent role. For this, lecturer feedback is also an important factor, feedback that leads to active student participation. The materials and the presentation of the content itself also play an important role, and it is essential to invite the participation and collaboration of the actors involved. Thus, the authors suggest the design of teaching strategies in which the student is an active actor (i.e., mentoring sessions, discussion dynamics in small groups) in the learning process to increase their involvement, motivation, and consequent level of engagement. The proposed model in this work will enable instructors to quantify the effects of these strategies in terms of students’ engagement.

The results obtained in this work are a starting point to continue this line of research and to continue contributing to how sensors can provide data for the study of student engagement in educational settings. In this regard, and taking into consideration the obtained results, the following are considered interesting future research directions.

First, it would be enriching to combine these results with those obtained in other studies. For example, the ontology proposed by Ayouni et al. [50], which comprises four dimensions (behavioral, cognitive, social and emotional), surely could provide new characteristics to be considered for their inclusion.

In reference to the model designed to quantify student engagement in synchronous VLEs, the authors believe that it is convenient to conduct a study to consider strengthening the model by including an automatic (and configurable) adjustment of the coefficients of the categories that takes into account the course of time in the session. In this way, the model presented would not only be automatically (and manually adjustable) according to the teaching activity, but would also take into account that, for example, five hands raised at the beginning of class may have a different meaning (i.e., impact in engagement) to if this occurred at the end of the session.

Finally, adding data related to students’ academic performance to those pertaining to student engagement could be enriching. Thanks to these rich data, it would be possible to apply machine-learning algorithms to discover hidden models that could be an advantage to, for example, design more efficient training activities, offer more personalized attention and detect academic dropout early [60]. In addition, their inclusion could be a starting point for calculating the level of individual engagement, taking into account not only digital features, but also the academic results.

## Figures and Tables

**Table 1:** Answers from the behavioral engagement category.

ID	Answer
B1	Interactions between teachers and students
B2	Participation to the activities and exercises proposed by the teacher (during lesson)
B3	Participation to project works proposed by the teacher (after lesson)
B4	Total duration of the microphone on for each participant
B5	Number of raised hands [52]
B6	Number of posts in meeting chat
B7	Number of screen sharing
B8	Number of interactions with student’s environment
B9	Percentage of students that have participated in tasks
B10	Lip movement time
B11	Number of interactions among the students and the teacher
B12	Answer to online form or pools
B13	Number of interactions among the students
B14	Number of student questions via chat
B15	Whether or not the camera is on
B16	Number of chat interactions
B17	Number of attendees
B18	Number of voice interventions

**Table 2:** Answers from the emotional category.

ID	Answer
E1	Type of stickers and emotions posted in chat
E2	Yawning
E3	Tone of voice in conversations
E4	Playing with hands, playing with hair, pens⋯
E5	Students not watching on the screen of the device
E6	Students talking with others
E7	Students switching off the camera
E8	Students watching around
E9	Non-verbal signs of appreciation, rejection and tension
E10	Student emotion
E11	Background noise
E12	Mean Opinion Score (MOS metric)
E13	Time in meeting
E14	Audio Discontinuity

**Table 3:** Answers from the cognitive engagement category.

ID	Answer
C1	Time to persevere with the task
C2	Number of answers which are: true/right/correct
C3	Number of correct answers to forms and pools
C4	Time to answer to online forms or pools
C5	Correct answers in debates/polls/surveys

**Table 4:** Answers identified as either implicit or out of scope.

ID	Answer	Reason
B1	Interactions between teachers and students	Implicit
B2	Participation to the activities and exercises proposed by the teacher (during lesson)	Implicit
B9	Percentage of students that have participated in tasks	Implicit
B3	Participation in project works proposed by the teacher (after lesson)	Out of scope
B12	Answer to online form or pools	Out of scope
E4	Playing with hands, playing with hair, pens…	Out of scope
E9	Non-verbal signs of appreciation, rejection and tensions	Out of scope
C1	Time to persevere with the task	Out of scope
C2	Number of answers which are: true/right/correct	Out of scope
C3	Number of correct answers to forms and pools	Out of scope
C4	Time to answer to online forms or pools	Out of scope
C5	Correct answers in debates/polls/surveys	Out of scope

**Table 5:** Resulting set of features obtained at the Feature Identification stage.

ID	Feature	Engagement
B4	Total duration of the microphone on for each participant	Behavioral
B5	Number of raised hands	Behavioral
B6	Number of posts in meeting chat	Behavioral
B7	Amount of screen sharing	Behavioral
B8	Number of interactions with student’s environment	Behavioral
B10	Lip movement time	Behavioral
B11	Number of interactions among the students and the teacher	Behavioral
B13	Number of interactions among the students	Behavioral
B14	Number of student’s questions via chat	Behavioral
B15	Whether or not the camera is on	Behavioral
B16	Number of chat interactions	Behavioral
B17	Number of attendees	Behavioral
B18	Number of voice interventions	Behavioral
E1	Type of stickers and emotions posted in chat	Emotional
E2	Yawning	Emotional
E3	Tone of voice in conversations	Emotional
E5	Students not watching on the screen of the device	Emotional
E6	Students talking with others	Emotional
E7	Students switching off the camera	Emotional
E8	Students watching around	Emotional
E10	Student emotion	Emotional
E11	Background noise	Emotional
E12	Mean Opinion Score (MOS metric)	Emotional
E13	Time in meeting	Emotional
E14	Audio Discontinuity	Emotional

**Table 6:** Result of grouping the 25 features obtained at the Feature Identification stage into 10 digital categories.

Digital Category	Engagement	No./% Total	Features
Attendance	Behavioral/Emotional	2/8%	(B17) Number of attendees, (E13) Time in meeting
Camera usage	Behavioral/Emotional	2/8%	(B15) Whether or not the camera is on, (E7) Students switching off the camera
Voice interactions	Behavioral	4/16%	(B4) Total duration of the microphone on for each participant, (B11) Number of interactions among the students and the teacher, (B13) number of interactions among the students, (B18) Number of voice interventions
Hand rising	Behavioral	1/4%	(B5) Number of raised hands
Screen sharing	Behavioral	1/4%	(B7) Number of screen sharing
Chat interactions	Behavioral/Emotional	4/16%	(B6) Number of posts in meeting chat, (B14) Number of student’s questions via chat, (B16) Number of chat interactions, (E1) Type of stickers and emotions posted in chat
Sound analysis	Behavioral	4/16%	(E3) Tone of voice in conversations, (E11) Background noise, (E12) Mean Opinion Score (MOS metric), (E14) Audio Discontinuity
Facial Emotion	Emotional	1/4%	(E10) Student emotion
Mouth-movement analysis	Behavioral/Emotional	3/12%	(B10) Lip movement time, (E2) Yawning, (E6) Students talking with others
Eye gaze	Emotional	3/12%	(E6) Students talking with others, (E5) Students not watching on the screen of the device, (E8) Students watching around
TOTAL		25/100%	

**Table 7:** Set of digital features defined for “Attendance” category, corresponding to features B17 and E13.

Level	Measurable Digital Feature
Group	(ATG1) Current number of attendees/number of enrolled students
Group	(ATG2) Maximum number of attendees/number of enrolled students
Group	(ATG3) Minimum number of attendees/number of enrolled students
Individual	(ATI1) Time in meeting

**Table 8:** Set of digital features defined for “camera usage” category, corresponding to features B15 and E7.

Level	Measurable Digital Feature
Group	(CUG1) Number of students/% of the time with camera on
Group	(CUG2) Number of students/% of the time with camera off
Group	(CUG3) Number of students/% of students that have switched the camera off once or more times
Individual	(CUI1) Current state (on/off) of the camera
Individual	(CUI2) Time camera on/% of the session elapsed time
Individual	(CUI3) Time camera off/% of the session elapsed time
Individual	(CUI4) Times that the camera has been switched off

**Table 9:** Set of digital features defined for the “voice interactions” category, corresponding to features B4, B11, B13 and B18.

Level	Measurable Digital Feature
Group	(VIG1) Overall spoken minutes by the lecturer/% of the session elapsed time
Group	(VIG2) Number of students that have spoken/% of the current attendees
Group	(VIG3) Overall spoken minutes by learners/% of the session elapsed time
Group	(VIG4) Overall silence minutes/% of the session elapsed time
Individual	(VII1) Whether or not has spoken
Individual	(VII2) Minutes spoken/% of the session elapsed time
Individual	(VII3) Times that has spoken

**Table 10:** Set of digital features defined for “Hand rising” category, corresponding to the feature B5.

Level	Measurable Digital Feature
Group	(HRG1) Number of students that have raised hand/% of the current attendees
Individual	(HRI1) Whether or not has raised hand
Individual	(HRI2) Number of times he has raised hand

**Table 11:** Set of digital features defined for “Screen sharing” category, corresponding to the feature B7.

Level	Measurable Digital Feature
Group	(SSG1) Number of students that have shared screen/% of the current attendees
Individual	(SSI1) Whether or not has shared the screen
Individual	(SSI2) Number of times he has shared the screen

**Table 12:** Set of digital features defined for “chat interactions” category, corresponding to features B6, B14, B16 and E1.

Level	Measurable Digital Feature
Group	(CIG1) Number of students that have written posts/% of the current attendees
Group	(CIG2) Number of posts written by students
Group	(CIG3) Type of stickers used
Individual	(CII1) Whether or not has written posts
Individual	(CII2) How many posts has written
Individual	(CII3) How many of those posts have been questions
Individual	(CII4) Type of stickers used

**Table 13:** Set of digital features defined for “facial emotion” category, corresponding to the features E10.

Level	Measurable Digital Feature
Group	(FEG1) Number of students for each of the emotions and % of the current attendees
Individual	(FEI1) Real-time main emotion

**Table 14:** Set of digital features defined for “sound analysis” category, corresponding to features E11, E12, E13 and E14.

Level	Measurable Digital Feature
Group	(SAG1) MOS
Group	(SAG2) Background noise
Group	(SAG3) Loudness
Group	(SAG4) Discontinuity

**Table 15:** Set of digital features defined for “mouth-movement analysis” category, corresponding to features B10, E2 and E6.

Level	Measurable Digital Feature
Group	(MMG1) Number of students that have yawned/% of the current attendees
Group	(MMG2) Number of students that have had interacted with other people outside the virtual session/% of the current attendees
Individual	(MMI1) How many times the student has yawned
Individual	(MMI2) Whether or not the student has had interaction with people outside the virtual session

**Table 16:** Set of digital features defined for “eye gaze” category, corresponding to features E6, E5 and E8.

Level	Measurable Digital Feature
Group	(EGG1) Number of students that have leaved the area captured by the camera/% of the current attendees
Group	(EGG2) Number of students that have been tagged as not watching at the screen at some moment/% of the current attendees
Individual	(EGI1) Times that the student leaves the area captured by the camera
Individual	(EGI2) Whether or not the student has not been watching at the screen⋯
Individual	(EGI3) Percentage of attention looking at the screen for each student

**Table 17:** Categorization of the identified features, 25 features classified in 10 digital categories.

Digital Category	Engagement	No./% Initial Features	No./% Digital Features
Attendance	Behavioral/Emotional	2/8%	4/9%
Camera usage	Behavioral/Emotional	2/8%	7/15%
Voice interactions	Behavioral	4/16%	7/15%
Hand rising	Behavioral	1/4%	3/7%
Screen sharing	Behavioral	1/4%	3/7%
Chat interactions	Behavioral/Emotional	4/16%	7/15%
Sound analysis	Behavioral	4/16%	4/9%
Facial Emotion	Emotional	1/4%	2/4%
Mouth-movement analysis	Behavioral/Emotional	3/12%	4/9%
Eye gaze	Emotional	3/12%	5/10%
TOTAL		25/100%	46/100%

**Table 18:** Relevance of each category according to its weight in the teaching activity.

	Lecture	Tutorial	Laboratory Class	Seminar	Doubts Session	PI
**Attendance**	VR	VR	VR	VR	VR	VR
**Camera usage**	I	I	I	R	R	VR
**Voice interactions**	R	R	R	R	R	R
**Hand rising**	R	R	R	R	VR	I
**Screen sharing**	I	I	R	I	R	I
**Chat interactions**	I	I	I	R	VR	I
**Sound analysis**	VR	SR	SR	VR	VR	VR
**Facial Emotion**	VR	VR	VR	VR	VR	VR
**Mouth-movement analysis**	VR	R	R	VR	R	VR
**Eye gaze**	VR	SR	SR	VR	SR	I

## Data Availability

Not applicable.
